# Gastrulation occurs in multiple phases at two distinct sites in *Latrodectus* and *Cheiracanthium* spiders

**DOI:** 10.1186/s13227-015-0029-z

**Published:** 2015-10-21

**Authors:** Allison Edgar, Christine Bates, Kay Larkin, Steven Black

**Affiliations:** Kleinholtz Biological Laboratories, Department of Biology, Reed College, 3203 S.E. Woodstock Blvd, Portland, OR 97202 USA; Department of Biology, Duke University, Durham, NC 27708 USA; Department of Internal Medicine, Duke University, Durham, NC 27708 USA

**Keywords:** Gastrulation, Morphogenesis, Arthropod, Chelicerate, Arachnid, Spider

## Abstract

**Background:**

The longstanding canonical model of spider gastrulation posits that cell internalization occurs only at a unitary central blastopore; and that the cumulus (dorsal organizer) arises from within the early deep layer by cell–cell interaction. Recent work has begun to challenge the canonical model by demonstrating cell internalization at extra-blastoporal sites in two species (*Parasteatodatepidariorum* and *Zygiella x*-*notata)*; and showing in *Zygiella* that the prospective cumulus internalizes first, before other cells are present in the deep layer. The cell behaviors making up spider gastrulation thus appear to show considerable variation, and a wider sampling of taxa is indicated.

**Results:**

We evaluated the model in three species from two families by direct observation of living embryos. Movements of individual cells were traced from timelapse recordings and the origin and fate of the cumulus determined by CM-DiI labeling. We show that there are two distinct regions of internalization: most cells enter the deep layer via the central blastopore but many additional cells ingress via an extra-blastoporal ring, either at the periphery of the germ disc (*Latrodectus* spp.) or nearer the central field (*Cheiracanthium mildei*). In all species, the cumulus cells internalize first; this is shown by tracing cells in timelapse, histology, and by CM-DiI injection into the deep layer. Injection very early in gastrulation labels only cumulus mesenchyme cells whereas injections at later stages label non-cumulus mesoderm and endoderm.

**Conclusions:**

We propose a revised model to accommodate the new data. Our working model has the prospective cumulus cells internalizing first, at the central blastopore. The cumulus cells begin migration before other cells enter the deep layer. This is consistent with early specification of the cumulus and suggests that cell–cell interaction with other deep layer cells is not required for its function. As the cumulus migrates, additional mesendoderm internalizes at two distinct locations: through the central blastopore and at an extra-blastoporal ring. Our work thus demonstrates early, cell-autonomous behavior of the cumulus and variation in subsequent location and timing of cell internalization during gastrulation in spiders.

**Electronic supplementary material:**

The online version of this article (doi:10.1186/s13227-015-0029-z) contains supplementary material, which is available to authorized users.

## Background

Spiders are an emerging system to probe arthropod development and the developmental origins of arthropod diversity [1]. As representatives of Chelicerata, the sister group to all other extant arthropod lineages [2], spiders are well positioned for comparative analysis. They are tractable in the laboratory, and much recent work has illuminated aspects of spider development such as axial (e.g. [3–5]), segmental (e.g. [6–8]), and regional patterning (e.g. [9, 10]). A relatively neglected area is the cell rearrangements driving gastrulation. Gastrulation is a key event in early development that converts the simple symmetry of the egg into the more complex symmetries of the later embryo. Subsequent morphogenesis builds on the outcome of gastrulation. While gastrulation varies widely across major taxonomic groups, it is not known whether modifications to gastrulation over smaller evolutionary time scales carry phylogenetic signal.

**Fig. 1:**
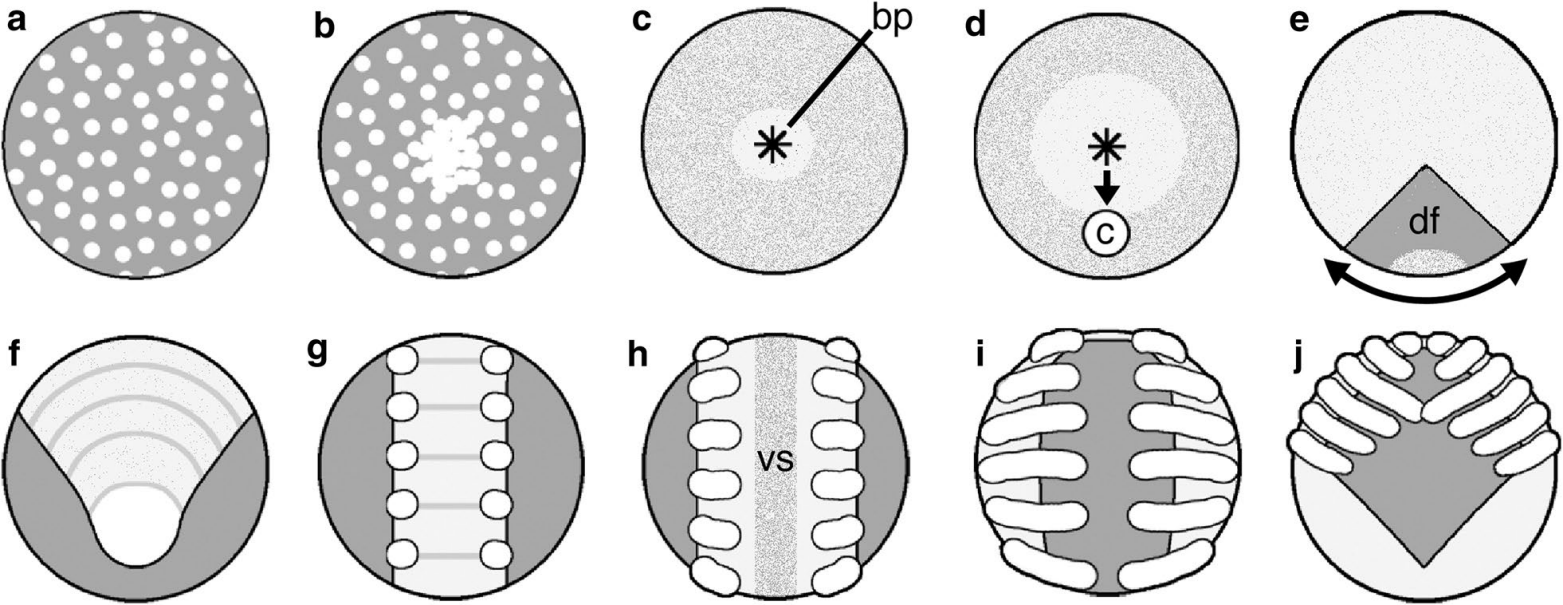
Canonical model of spider development, external view. **a** Blastoderm forms. **b** Blastoderm cells proliferate and migrate to one hemisphere to form a germ disc. **c** Primitive plate forms by internalization at a central blastopore. *Asterisk* marks blastopore (bp). **d** Cumulus (c) originates in the deep layer near blastopore and migrates radially to the prospective dorso-posterior side of the germ disc. **e** Germ disc cells move away circumferentially from the cumulus endpoint. This movement forms the germ band; the thinned area is termed the dorsal field (df). **f** Segmentation becomes apparent in the germ band. **g** Limb primordia appear on the anterior six segments (the prosoma). **h** The germ band splits along the mid-sagittal plane to form the ventral sulcus (vs). **i** The two halves of the germ band move laterally around the yolk, a process called inversion. **j** The prosoma condenses on the dorso-anterior side of the embryo and a sheet of cells surrounds the yolk. Additional file 1: Figure S1, Additional file 2: Figure S2 and Additional file 3: Figure S3 show photographs and timing of events in normal development of the species studied here

The current ‘canonical model’ of spider gastrulation (summarized in Fig. 1) emerged largely from Holm’s excellent embryological work on the labyrinth spider, *Agelena labyrinthica* [11–13]. *A. labyrinthica* has been a longstanding model for spider development [14, 15]. The model has been elaborated but substantially unchallenged by modern studies of gastrulation using the common house spider, *Parasteatodatepidariorum* (previously *Achaearanea* [16]) [17, 18]; reviewed in [19] and the wandering spider, *Cupiennius salei* (e.g. [20, 21]).

Spider development begins as early cleavage nuclei migrate from the interior to form a monolayered blastoderm that evenly covers the yolk. Despite a superficial resemblance to the *Drosophila* syncytial blastoderm, spider embryos exhibit a form of total cleavage from at least the 16-cell stage, as demonstrated by three lines of evidence: older histological work described ‘yolk pyramids’ suggestive of yolk compartmentation [22]; injected fluorochrome-conjugated dextran does not diffuse beyond these compartment boundaries in *P. tepidariorum* [23]; and similar pyramidal compartments appear in SEM of fractured *C. salei* embryos [21]. In some species, most of the blastoderm cells migrate towards one hemisphere to form a distinct germ disc. At these early stages, the geometry of the spider embryo is analogous to that of the chicken, in that the embryo arises from a thin disc of cells resting on a larger yolk mass.

Gastrulation begins near the center of the radially symmetrical germ disc (Fig. 1). As cells internalize, the multilayered portion of the germ disc appears opaque and is commonly termed the ‘primitive plate’ or ‘primary thickening’ [14, 24]. Two distinct populations of internalized cells compose the deep layer in every spider embryo studied to date: a dorsal organizer termed the ‘cumulus’ and a presumably mixed population of prospective mesoderm and endoderm cells (mesendoderm). The canonical model of spider development asserts that these two cell populations become specified only after significant internalization of a deep layer.

The cumulus is a small group of mesenchymal cells that actively migrates to the prospective dorso-posterior edge of the germ disc. The arc defined by the blastopore (posterior) and the cumulus’s endpoint (dorsal) effectively implies all body axes. The cumulus is necessary and sufficient to establish the body axes: surgical extirpation results in radialized embryos and ectopic cumulus implantation duplicates the body axis [11, 19]. Furthermore, its cells express *decapentaplegic* (*dpp*) mRNA [17], and knockdown of *dpp* results in severe axial defects including radialization of the dorsal–ventral axis [18]. The majority of cells in the deep layer is not part of the cumulus and will form the bulk of the mesoderm and endoderm. In fixed embryos, the cumulus is morphologically distinct. Seen by scanning electron microscopy, the cumulus deep cells of *P. tepidariorum* appear almost spherical [17]. In histological sections from other species, their appearance is similar: cumulus cells are large, round, and often vacuolated or relatively lightly stained [25, 26].

Cell rearrangements transform the original disc into an elongated germ band as the cells along the cumulus’s path spread out. The newly thinned area is termed the dorsal field, and will form extraembryonic tissues. The multilayered area (light color in Fig. 1e) comprises many more cells and will form the germ band. The germ band then splits longitudinally along the ventral midline to form the ventral sulcus and its two halves (the right and left sides of the body) migrate to opposite sides of the yolk. This process is called inversion, and occurs in most spiders and one other arachnid order [27]. Subsequent ventral and dorsal closure movements complete the spiderling.

The canonical model of gastrulation is valuable because it provides a shared framework for research; however, variation from this model among spider species would indicate that the evolution of different gastrulation strategies can happen over smaller time scales. Furthermore, there remain important unanswered questions about the basics of spider gastrulation. There are two questions that interest us most: (1) Is the central blastopore the only point of cell internalization? Multiple sites of internalization would violate the canonical model, and the data in the literature are somewhat contradictory, see below. (2) What is the timing of internalization of the cumulus cells relative to the rest of the deep layer? This is interesting from the point of view of cell specification: if the cumulus arises before other cells internalize, then it is likely to be already specified rather than forming as a result of cell–cell interaction within the deep layer.

Whether there are multiple sites of gastrulation in spiders has been a point of contention in historical and contemporary literature. Histological, molecular, and lineage tracing evidence support cell internalization at both the center and the peripheral rim of the germ disc. Rempel showed drawings of histological sections with an apparent accumulation of cells at the rim of the germ disc in the black widow spider, *Latrodectus mactans* [28], which confirmed earlier work by Montgomery [22]. In *P. tepidariorum*, *twist*-expressing cells in the deep layer were shown to originate at the periphery of the germ disc [29]. However, Wolff and Hilbrant [21] found no evidence of internalization at the germ disc rim in *C. salei*. In the silver-sided sector spider, *Zygiella x*-*notata*, Chaw et al. [26] used timelapse video to document cell behavior of the superficial cells of the germ disc and likewise found no internalization at the germ disc rim. To evaluate whether or not internalization at extra-blastoporal sites is a general phenomenon of spider development, cell tracing in living embryos is required. Static observations alone—histological or gene expression patterns—cannot reveal the spatial origin of actively migrating cells. For example, deep cells at the rim of the germ disc could arise from the central blastopore and migrate to the periphery [21].

For this paper, we used high-resolution timelapse videography, direct labeling of cells with CM-DiI, and improved histology to evaluate gastrulation in three spider species. In *Latrodectus* spp. (*L. mactans* and *L. geometricus*, the brown widow spider) and in the yellow sac spider, *Cheiracanthium mildei*, the cells of the cumulus internalize early and separately from the primary mesendoderm. We document active internalization at the rim of the germ disc in *Latrodectus* spp. and in a region outside the central blastopore in *C. mildei*. The timing of cumulus formation is consistent with a cell-autonomous mechanism for its formation. Our results provide the most detailed evaluation of cell behavior during gastrulation yet achieved in spiders, and suggest modifications to the canonical model.

## Methods

*Organisms* Adult *Cheiracanthium mildei* and their egg sacs were collected locally. Females were housed individually at room temperature (22°–24 °C) with a 12-h light cycle, and fed crickets to satiation. Spiders were occasionally mated in the lab. Egg masses contained about 30–100 eggs, each 0.7–0.9 mm in diameter. Adult *Latrodectus mactans* and *Latrodectus geometricus* were purchased from SpiderPharm and were housed and fed similarly to *C. mildei*. The spiders were mated immediately before shipping by SpiderPharm, for which we are very grateful. Most egg sacs contained over 70 eggs, each 0.7–1.0 mm in diameter. Embryos of both species were incubated at room temperature (22–24 °C), or at 18 °C to retard development.

*Time*-*lapse imaging and tracing* Embryos were immersed in mineral oil (Sigma) to clear the chorion. Time-lapse frames were captured every 3–5 min using Astro IIDC software running on Macintosh computers. Prisms were sometimes used to image the blastoporal region. Using Adobe Photoshop, all frames were cropped and adjusted for brightness and contrast; a high-pass sharpening filter was used to sharpen cell borders in some movies. Batch-processing was automated for consistency and convenience. Individual cells were traced by hand on individual frames.

*CM*-*DiI injection* CM-DiI (1,1′-dioctadecyl-3,3,3′,3′-tetramethylindocarbocyanine perchlorate) (Invitrogen) is a fixable DiI. It was diluted in 16 % DMSO in 80 % ethanol in PBS and used at 170 μg/mL. In *L. mactans*, 0.5 nL was injected, and in *C. mildei*, up to 2 nL was injected. Embryos were periodically photographed under white light and under 560 nm excitation.

*Fixation C. mildei* embryos at various stages were collected and treated with Ilsa (58 % methanol, 17 % chloroform, 17 % DMSO, 8 % acetic acid; made fresh, see [26]). Embryos were stepped into 100 % methanol, and stored at −20 °C. Embryos were postfixed by stepping into room temperature PBS, transferred into 4 % paraformaldehyde in PBS made fresh and manually demembranated using tungsten needles and watchmaker’s forceps. Demembranation was completed in 0.1 % Triton X-100 in PBS as necessary to ensure that total time in paraformaldehyde did not exceed 30 min.

*Latrodectus mactans* embryos were dechorionated using a 50 % percent bleach solution and fixed in Ilsa until opaque. Glass or tungsten needles and watchmaker’s forceps were used to remove the vitelline membranes in one of various postfixation formulas, see below.

For histology, postfixation was carried out in Nu–Nu Fix (4 % paraformaldehyde, 4 % glacial acetic acid, 1 mM each calcium chloride and magnesium sulfate in PBS) for 30–60 min. In some cases, the embryos were fixed for 48 h and then postfixed in 1 % paraformaldehyde in PBS for 5 days. For immunostaining, Nu–Nu Fix + 5 % DMSO was used for 15 min maximum. After postfixation, embryos were stepped into methanol for histology or storage at −20 °C, or were moved directly onto antibody staining.

*Embedding and sectioning* To aid in orientation, postfixed embryos were pre-stained with Eosin B-Phloxine (3 drops 0.1 % stock in 10 mL methanol) or Delafield’s hematoxylin in methanol in the same proportions. They were stepped into paraffin via graded series of methanol, tert-butyl alcohol, and mineral oil. Blocks were sectioned until tissue was encountered and soaked overnight in 5 % glycerol. Paraffin sections 6–8 μm were mounted with degassed Mayer’s albumen and stained with Delafield’s Hematoxylin and Eosin B-Phloxine; or Delafield’s Hematoxylin and Eosin B-Phloxine + 0.02 % Fast Green for *L. mactans*; or with Nuclear Fast Red + Orange G + 5 % Phosphotungstic acid + Black’s Aniline Blue Orange G (spiderlings). DAPI 1:1000 was used as a counterstain for some CM-DiI-injected specimens. Sections were coverslipped with Pro-Texx Mounting Medium (Baxter Diagnostics).

*Antibody staining and confocal microscopy* Embryos were stepped into PTD (0.1 % Triton + 5 % DMSO in PBS), washed 5X in PTD, blocked 30 min in 5 % normal goat serum + 5 % DMSO + 0.02 % sodium azide in PTD. Embryos were stained overnight at room temperature in 1:1000 mouse anti-tubulin (Sigma) in blocking solution and washed and blocked as above. Embryos were incubated overnight at room temperature in 1:1000 Alexa-conjugated goat anti-mouse (Invitrogen) in blocking solution and then washed again and stepped into methanol. To stain nuclei, embryos were incubated overnight at 4 °C in 1:1000 Yo-Pro-1 in methanol, washed 3 times in methanol, and cleared with methyl salicylate. Images were acquired with an Olympus Fluoview confocal microscope and stacks were compiled in Image J (NIH.gov).

## Results

**Fig. 2:**
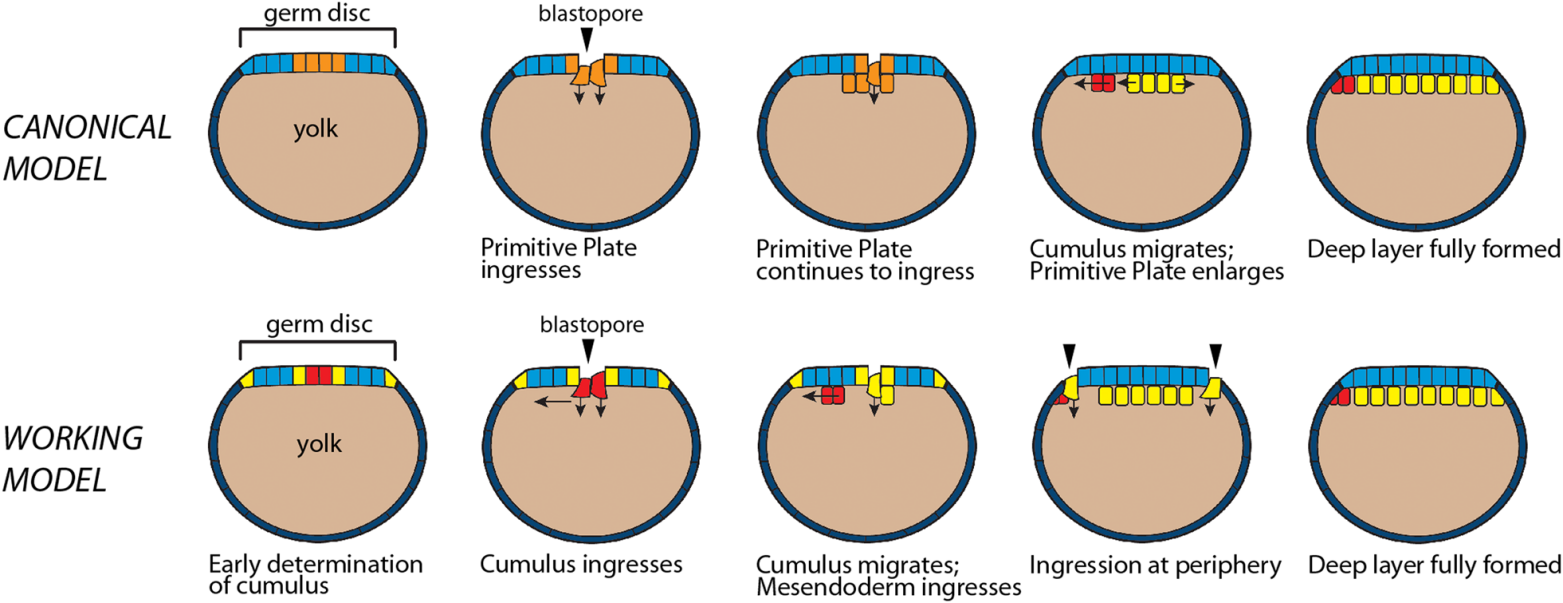
Proposed revisions to the canonical model of spider gastrulation. Diagrammatic midsagittal sections. Ectoderm is *blue*. Top row: *Canonical model.* A mixed population of cumulus cells and deep layer mesendoderm cells (*orange*) enters through the central blastopore. The cumulus (*red*) then differentiates and migrates towards the prospective dorsal side as the primitive plate (*yellow*) enlarges. Gastrulation ends when a deep layer has formed under the entire germ disc and signaling from the cumulus has broken radial symmetry. Bottom row: *Our working model.* The cumulus mesenchyme cells (*red*) internalize first, as a group, through the central blastopore. As the cumulus migrates, additional mesendoderm (*yellow*) cells internalize at two distinct locations: through the central blastopore and at the rim of the germ disc or, in species lacking a germ disc, a region several cells away from the central blastopore

In all three species studied, we show that the cells of the cumulus internalize first and in concert. There is then a pause in the internalization process, after which non-cumulus mesendoderm cells begin to internalize. There is variation between the two *Latrodectus* species versus *C. mildei* in the location of this second round of internalization. In *Latrodectus* spp., mesendoderm internalizes along a linear blastoporal region that includes the former site of cumulus ingression. Much later, after cumulus migration is complete, cells also internalize in situ at the rim of the germ disc. In contrast, secondary internalization in *C. mildei* occurs in situ at a ring or region approximately two–five cell lengths away from the original blastopore. Thus, in both genera, cells internalize first at a central blastopore (to form the cumulus) and subsequently at other positions on the blastoderm (to form general mesendoderm), see Fig. 2. The early timing and unitary site of internalization for the prospective cumulus strongly suggest that the cells of the cumulus are already committed to their fate as the dorsal organizer when they internalize. The canonical model’s assumption that the cumulus arises from a subpopulation of generalized primitive plate cells within the deep layer is not consistent with what we observe in the species studied here, as virtually all the early ingressing cells form the cumulus.

### *Latrodectus mactans* and *L. geometricus*

#### Timing of cumulus internalization and its fate

**Fig. 3:**
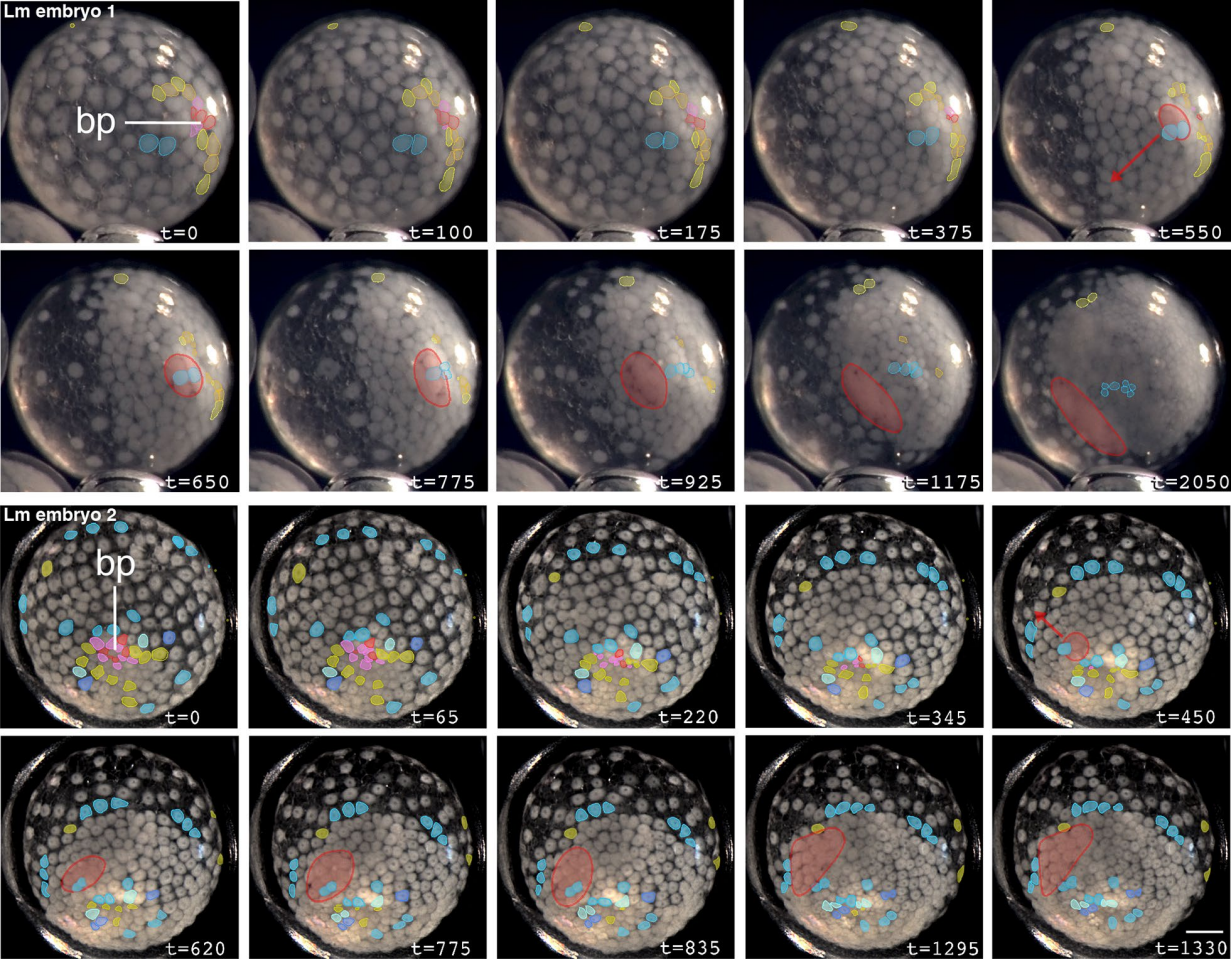
Gastrulation proceeds in two phases in *L. mactans* embryos. Tracings of external views of living embryos show that prospective cumulus cells (*red* and *pink*) internalize first at the blastopore (bp). Internalized cumulus cells are *circled* in *red* and migrate along *red arrow*. Other presumptive lower layer cells (*shades of yellow*) internalize later to form generalized mesendoderm. Note *yellow* cells at periphery of germ disc; these were traced and will internalize later. *Blue* cells are those known to persist in the superficial layer of the germ disc as long as their daughter cells can be visualized. Times in min. *Scale bars* 200 µm. Timelapse movies of Lm embryos 1 and 2 are shown in Additional file 4: Movie 1 and Additional file 5: Movie 2)

In *Latrodectus* spp., the presumptive cumulus cells are the first cells to internalize. We show this in two ways: by virtual tracing of cells in timelapse movies and by direct labeling of presumptive cumulus cells with CM-DiI. Tracings of two representative *L. mactans* embryos are shown in Fig. 3, in which the first cells to enter the deep layer at a central point blastopore are colored red and pink. Immediately after most of these cells internalize, even while a few are still visible at the blastopore, the cumulus begins to migrate. It can be seen as a prominent bulge beneath the germ disc originating below the blastopore (red outline, Fig. 3). During this early phase of cumulus migration, there is a distinct pause of about 1 h while no further cells are internalized. Then internalization resumes at the same blastoporal region as non-cumulus cells begin to ingress. At this stage, the blastopore elongates to form a groove (most easily seen in Lm embryo 1—note slight depression at times 550–1175 min, Fig. 3; Additional file 4: Movie 1); and non-cumulus mesendoderm internalizes (yellow cells). Internalization appears to be somewhat asymmetric; more cells internalize on the side of the groove away from the path of cumulus migration (this can be seen qualitatively in timelapse movies). Much later, after the cumulus has migrated, additional mesendoderm ingresses at the rim of the germ disc (discussed in more detail below). We were unable to produce tracings in *L. geometricus* to confirm the same result in that species, but our histological data (see below) are consistent with the view that the cumulus cells arise by a conserved process in both *Latrodectus* species.

**Fig. 4:**
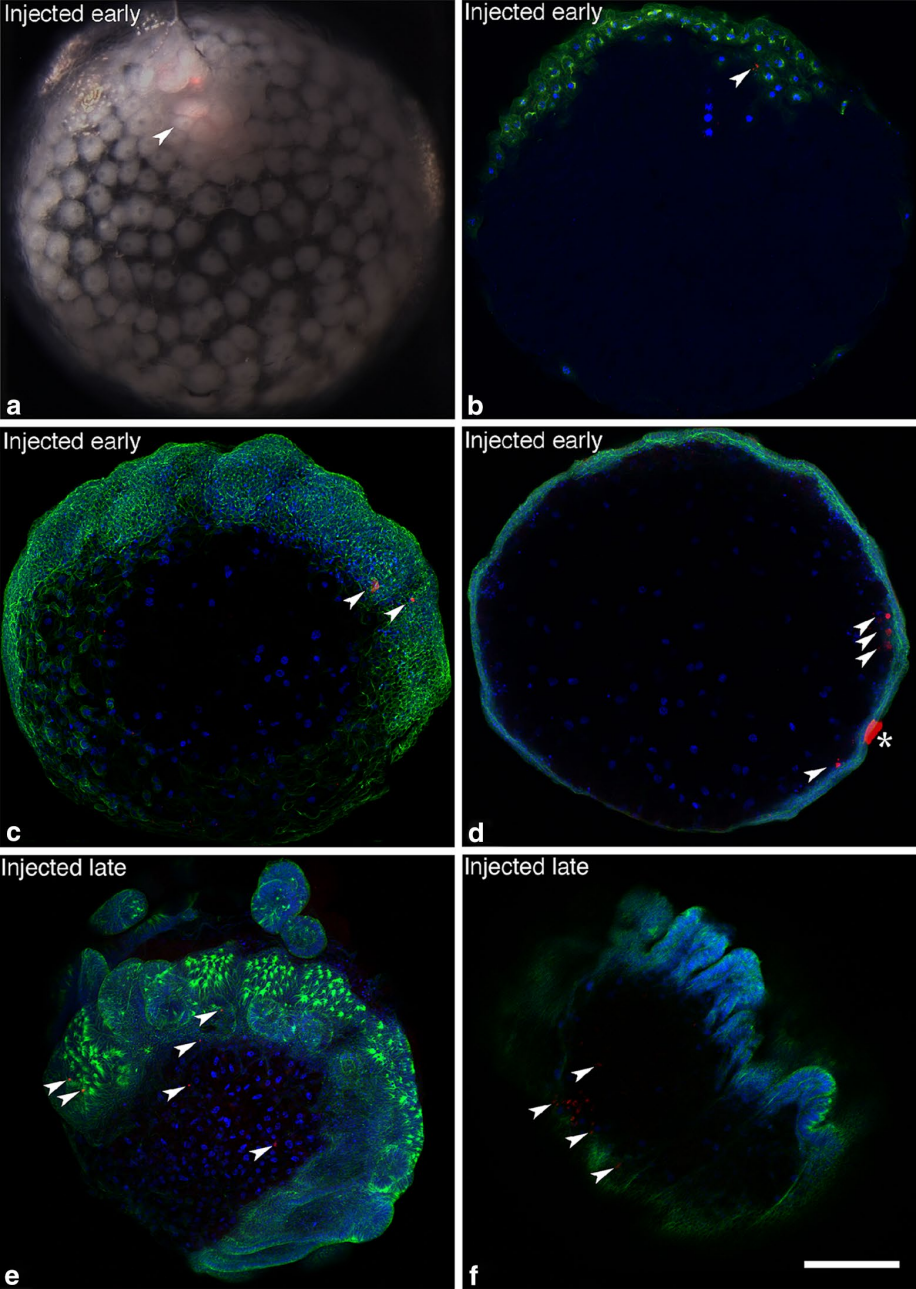
Direct labeling of the deep layer confirms that cumulus cells internalize first in *L. mactans*. **a** External view just after CM-DiI injection through blastopore into early deep layer, *arrowhead* indicates label (*red*) in cumulus. **b** Confocal section of similar embryo fixed 1 day after injection; red dye is limited to cumulus mesenchyme (*arrowhead*). A few cells appear to have been pushed deeper into the embryo during injection. Tubulin is *green*, nuclei are *blue*, *arrowheads* mark labeled cells in **b**–**f**. **c**, **d** Injection into early deep layer. **c** Confocal stack of embryo fixed 1 week after injection; label is in posterior germ band consistent with normal migration path of cumulus. **d** Confocal section of a similar embryo fixed 1 week after injection; labeled cells are in deep layer at posterior. *Asterisk* marks fluorescent debris attached to exterior of embryo. **e**, **f**. Injection into later deep layer below blastopore, after cumulus has migrated away from blastopore (~1 day later than early injection). **e** Confocal stack of embryo fixed 2 weeks after injection; labeled cells are in right germ band and extraembryonic area. **f** Confocal section of similar embryo fixed 2 weeks after injection; labeled cells are in deep layer of right germ band. Position of label is consistent with non-cumulus fate. *Scale bars* 200 µm

To confirm that the first cells to internalize are prospective cumulus cells, we microinjected the cell tracer CM-DiI into the deep layer cells immediately below the blastopore. We then traced the path of migration of the first ingressors and determined their fate later in development. When injected early in gastrulation, before cumulus migration has started, the label was restricted to the cumulus during its migration. No labeled cells appeared in any other region of the germ disc 5–16 h post-injection, showing that the first cells to internalize contribute exclusively to the cumulus (*N* = 24, 12 followed to germ band stage; Fig. 4a, b). Subsequent position of the label is consistent with what is known about the fate of the cumulus once its cells disperse ([11], this paper); labeled cells were found in the posterior germ band 9–14 days after injection (*N* = 12; Fig. 4c, d). In contrast, injection below the blastopore after the cumulus was in migration labeled only non-cumulus cells (i.e., no later-ingressing cells joined with the cumulus). After 14 days, labeled cells in these embryos were found in the deep layer of the lateral germ bands (except the appendages) and extraembryonic areas (*N* = 6; Fig. 4e, f). These locations are consistent with a generalized ‘mesendodermal’ fate and differ from the solely posterior position of the dispersed cumulus cells. Thus two approaches to the problem of the timing and spatial origin of the cumulus cells yield the same answer: all and only prospective cumulus cells internalize first and in concert, before generalized mesendoderm enters the deep layer. This suggests that prospective cumulus cells become committed to their fate without the requirement for cell–cell interaction within the deep layer, as cumulus migration begins before other cells have internalized.

#### Histology confirms that the cumulus cells internalize before other cell types

**Fig. 5:**
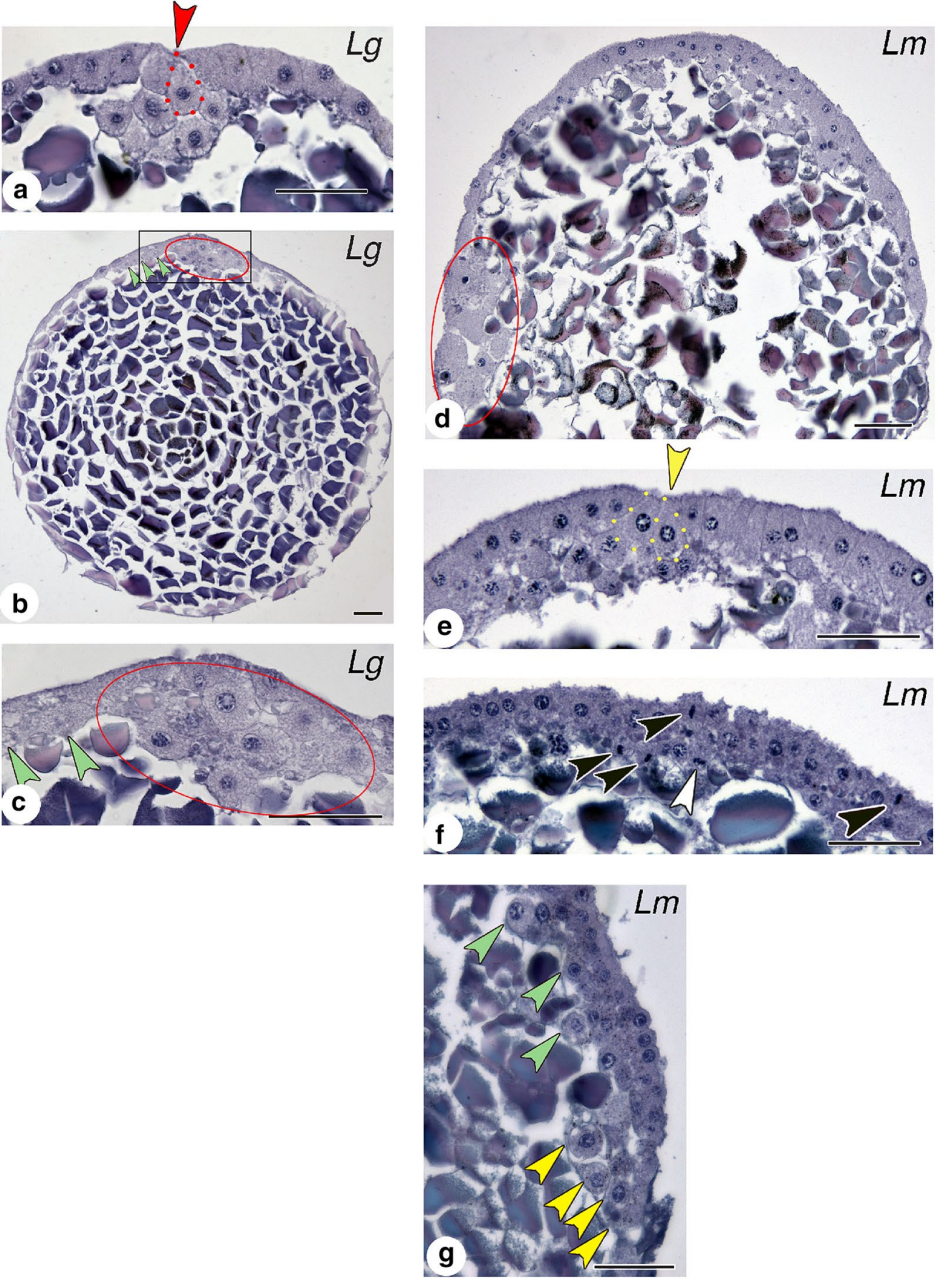
Cell morphology at two loci of internalization in *Latrodectus species.* **a** Beginning of gastrulation: presumptive cumulus cells internalize through central blastopore (*arrowhead*). Bottle cell outlined. *L. geometricus.* **b** Slightly later stage: most deep cells are cumulus mesenchyme (*circled*). Cumulus has begun to migrate. A few primitive plate cells have ingressed (*green arrowheads*). *L. geometricus.* **c** Closeup of boxed area in **b**. **d** Mid-gastrula: cumulus (*circled*) has migrated to edge of germ disc. Blastopore is not in plane of section. *L. mactans.* **e**. Late gastrula: primitive plate comprises two to three layers of deep cells. Internalization continues at central blastopore (*arrowhead*). Two bottle cells outlined. *L. mactans.* **f** Mitotic spindles are oriented such that daughter cells typically remain in the same cell layer (*black arrowheads*), although some cell divisions are vertical (*white arrowhead*). *L. mactans.* **g** Section through rim of germ disc late in gastrulation showing a chained array of large, round cells (*yellow arrowheads*). *Green arrowheads* indicate some mesenchymal cells in the deep layer. *L. mactans. Scale bars* 50 µm

Histology can reveal details of cell shape diagnostic of cell type and from which cell behaviors can be inferred [30]. In *Latrodectus* spp., the cumulus cells are typically larger and more spherical than adjacent cells. Sections of early *L. geometricus* embryos show that the presumptive cumulus cells ingress first (Fig. 5a): no other deep cells are found in serial sections. When internalization of other primitive plate cells begins, the cumulus is seen as a mass of spherical cells distinct from the other cells of the deep layer, which are spindle-shaped at this early stage (Fig. 5b, c). In both species, internalizing cumulus cells and other primitive plate cells show a bottle cell shape, where a cell is apically constricted with a wide basal region (e.g., outlined cells in Fig. 5a, e). This is indicative of invagination or ingression in a wide variety of taxa [31]. After internalization, most non-cumulus deep cells appear mesenchymal whereas columnar and cuboidal epithelial cells make up the germ disc surface (Fig. 5d–g). If non-cumulus cells appear round, they are distinctly smaller than those composing the cumulus (e.g., Fig. 5d). Rempel [28] reported some vertical mitoses contributing to the formation of a deep layer, but our sections confirm Rempel’s view that most mitoses in the blastoderm are horizontal rather than vertical (Fig. 5f). Such horizontal orientation of mitosis could contribute primarily to the epibolic expansion of the superficial layer rather than adding cells to the deep layer.

#### Evidence for internalization at the germ disc rim

**Fig. 6:**
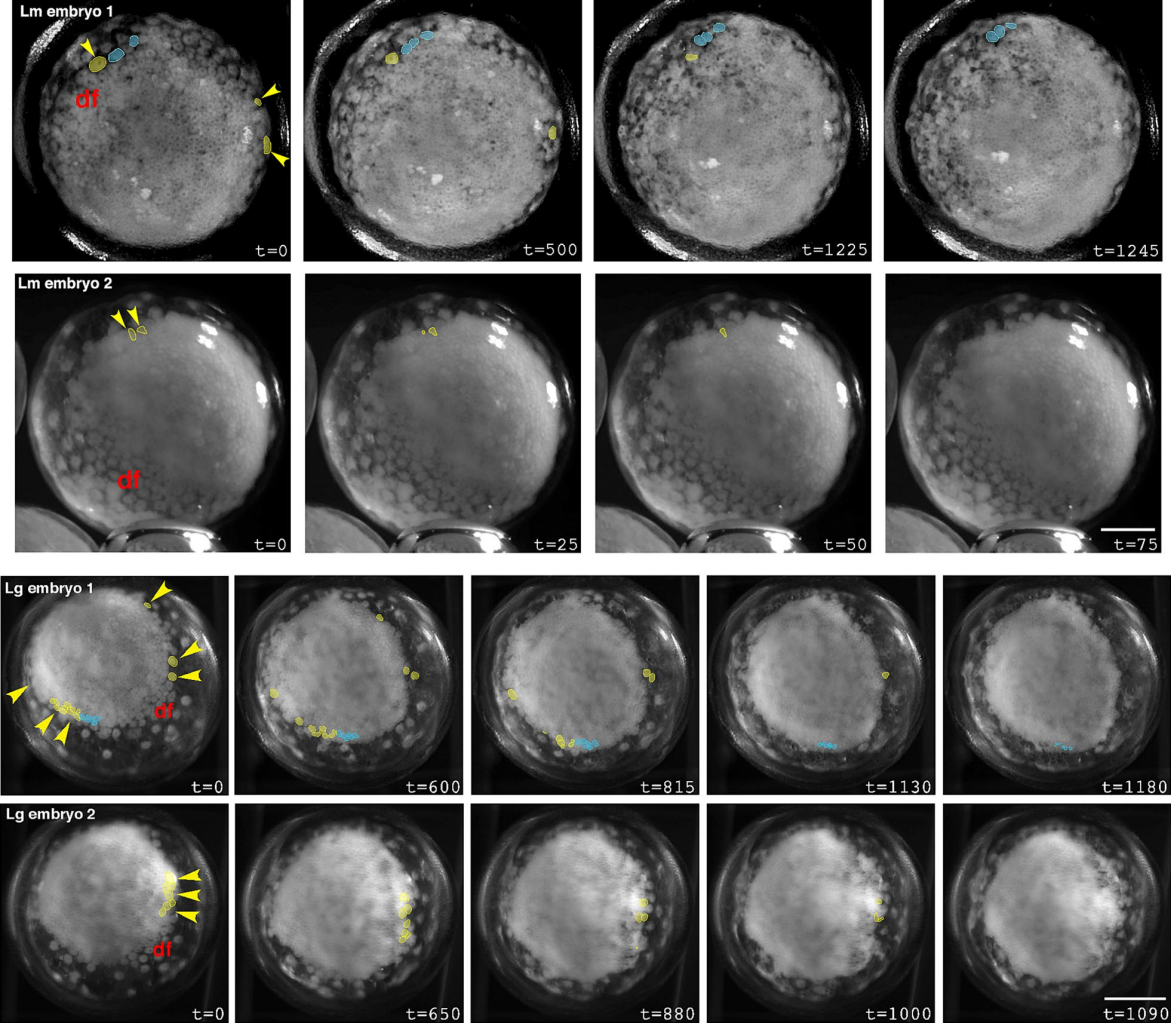
Cells internalize at the rim of the germ disc in *Latrodectus* species. External views of late gastrulae show internalization of prospective mesendoderm (*yellow*) and, in *blue*, examples of cells known to persist in the upper layer of the germ disc as long as their daughter cells can be visualized. *Arrowheads* point to cells that will internalize. df, dorsal field, region to which cumulus has migrated. Lm, *L. mactans*. Lg, *L. geometricus.* Times in min. *Scale bars* 200 µm. Timelapse movies of Lm embryo 1 and Lg embryo 1 are shown in Additional file 6: Movie 3 and Additional file 7: Movie 4

Our tracings of cells in timelapse videos during late gastrulation show definitively that cells internalize at the rim of the germ disc in both *L. mactans* and *L. geometricus* (Fig. 6; Additional file 6: Movie 3, Additional file 7: Movie 4). Internalization at the rim begins about 14 h after gastrulation at the central blastopore has ceased. At this stage, the cumulus has completed migration and the dorsal field has begun to form. In *L. mactans* internalization at the rim appears to contribute only a few cells to the deep layer.

Round cells of intermediate size are visible in the deep layer of histological sections late in gastrulation (Fig. 5g, green arrowheads). In sections of *P. tepidariorum*, Montgomery [22] notes apparent cell internalization around the rim of the germ disc, and in *L. mactans* Rempel [28] notes a chain of deep-layer cells linked in series and connected to the superficial layer at the germ disc rim. Our sections show virtually the same static image as presented by Rempel (Fig. 5c *cf.* his Fig. 26), and is compatible with internalization at the rim of the germ disc, perhaps by involution. This is consistent with the expression of *At*-*twist* and *fkh* at the rim of *P. tepidariorum* [18, 32]. However, the problem shared by histology and static gene expression patterns is that such studies cannot reveal the origin of the cells; for example, in the case of *At*-*twist* and *At*-*fkh*, cells expressing these markers are found at the rim but may have originated at the central blastopore and simply migrated. In *P. tepidariorum*, this issue was ultimately addressed by following a labeled clone in timelapse video, to obtain direct evidence of internalization at the rim [29]. In *Latrodectus*, we followed the movements of individual cells at the rim in video recordings.

Although the chained array of cells seen in sections was suggestive of involution, our videos show no mass movements or inrolling—internalizing cells left the surface only by ingression. This pattern of individual ingression is also seen in embryos of *L. geometricus*, with more cells internalizing at the rim than in *L. mactans* (Fig. 6). As with *L. mactans*, *L. geometricus* cells internalize at multiple positions around the rim of the germ disc. We did not see internalization of cells in the central germ disc at this stage in timelapse movies of either species, and neither did we see bottle cells in the central region at these later stages in our extensive histological series.

### *Cheiracanthium mildei*

#### Central and ring blastospores are distinct sites of internalization

**Fig. 7:**
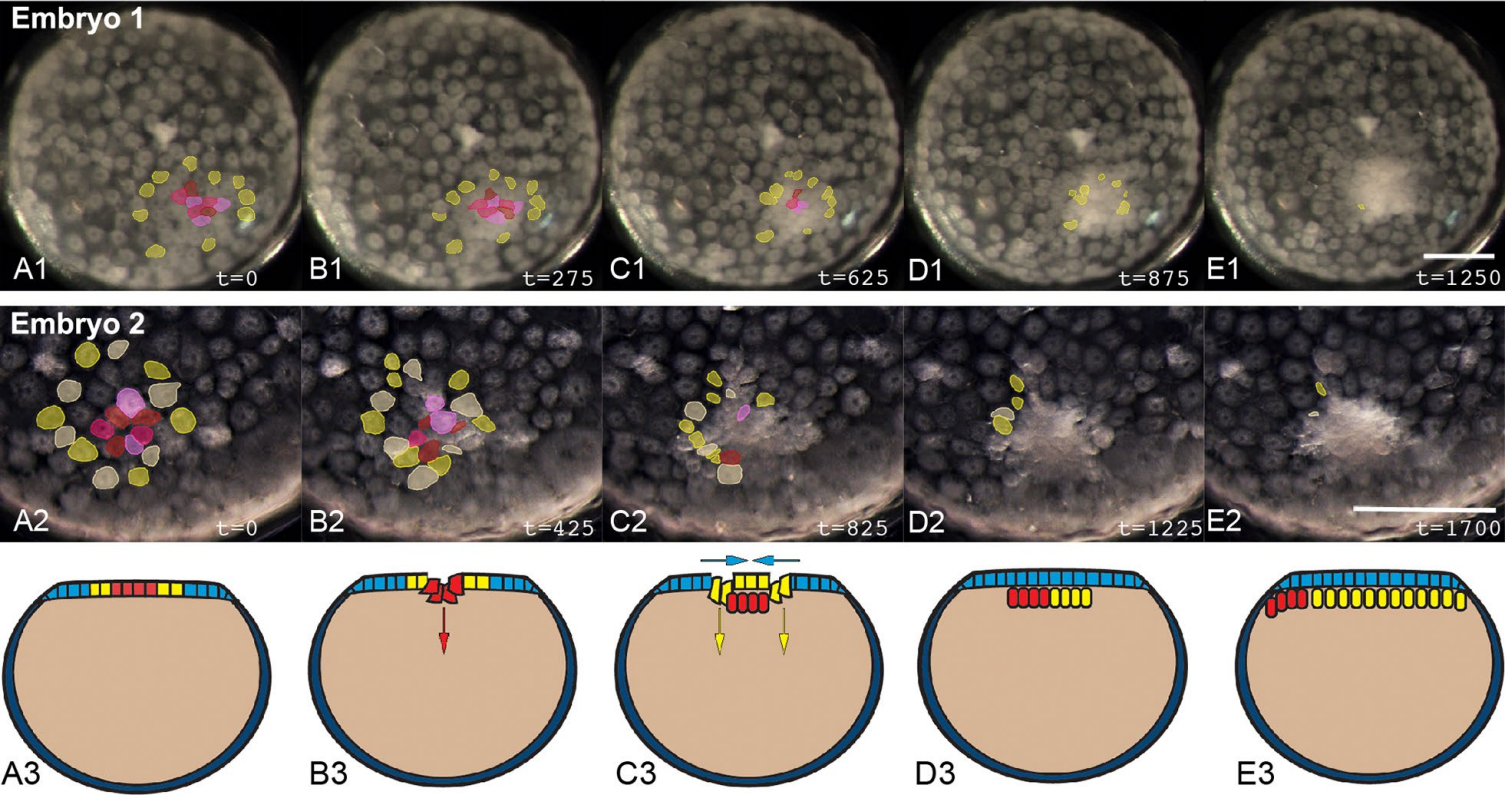
Gastrulation at two distinct loci in *Cheiracanthium mildei*. *A1–E1, A2–E2* External view of two representative embryos show internalization of different cell populations at two distinct loci. First, cumulus cells (*red* and *pink*) internalize through a central blastopore; then generalized mesendoderm (*yellow*) internalizes in situ at a separate ring outside the blastopore. Some mesendoderm likely internalizes at the original blastopore as well, see text. Times in min. *Scale bars* 200 µm. Timelapse movie of Cm embryo 1 is shown in Additional file 8: Movie 5. *A3–E3* Representation of gastrulation in *C. mildei* from our timelapse and histological data (not shown). Diagrammatic midsagittal sections. Ectoderm is *blue*, cumulus is *red*, mesendoderm is *yellow*

Similar to many spiders (*Ischnothele karschi* [12], *C. salei* [21]), *C. mildei* embryos lack a sharply defined boundary at the edge of the germ disc. Nevertheless, at germ disc stage the majority of cells occupy one hemisphere of the embryo, and the blastopore is located near its pole. Frame-by-frame cell tracings of timelapse movies of *C. mildei* show that early gastrulation is similar to that of other species: the cumulus cells internalize as a group through the central blastopore (Fig. 7; Additional file 8: Movie 5), as in *L. mactans* (this paper), *Z. x*-*notata* [26], and *P. tepidariorum* (our unpublished observations). However, internalization of the cumulus is followed approximately 3 h later by a novel gastrulation process: cells ingress in a ring approximately 2–5 cell lengths from the original blastopore. The new region of ingression is spatially distinct from the point through which cumulus cells internalized, although some cells are adjacent (e.g. Embryo 2 in Fig. 7). Cells internalizing via the ring probably contribute mesendoderm to the primitive plate. It seems likely that additional mesendoderm also internalizes at the original blastopore at this stage, but the central cells are so tightly packed that they cannot be resolved individually. Because the number of cells seen to internalize at the annular blastopore does not account for the entire lower layer in *C. mildei*, it is likely that both areas continue to internalize mesendoderm during the second round of gastrulation.

**Fig. 8:**
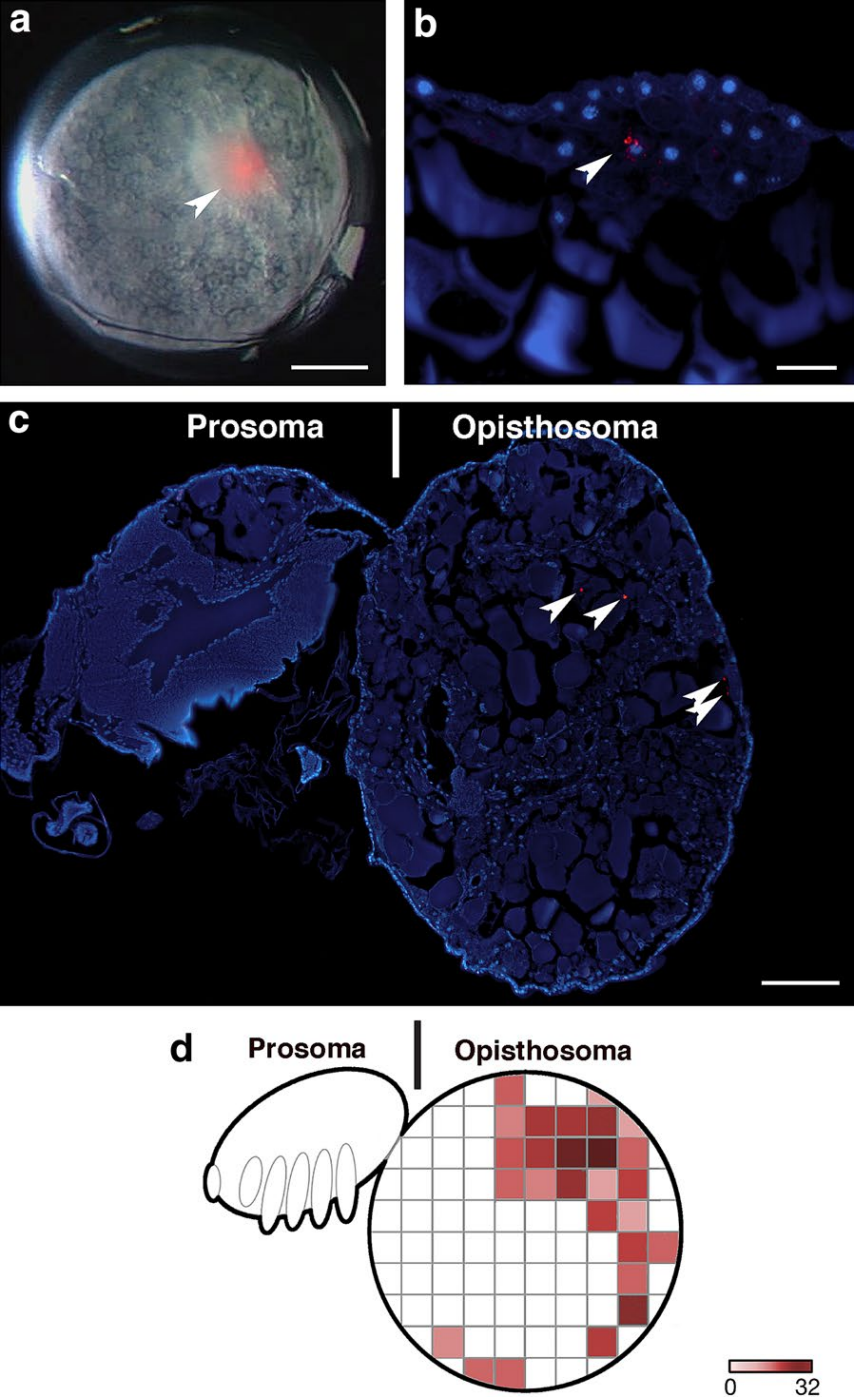
The cumulus internalizes first and its cells have an opisthosomal fate in *C. mildei*. **a** Early gastrula soon after injection of CM-DiI (*red*) into the early deep layer below the blastopore. **b** Representative vertical section (10 µm, paraffin) through cumulus 6 h after CM-DiI injection. Nuclei stained with DAPI. **c** First-instar juvenile 10 days after injection, parasagittal section near midline. *White arrowheads* indicate label. **d** Heat map showing positions of label in serial sections spanning ~160 µm of the sagittal midline. Counts were summed for four juveniles with a maximum value of 32 points of label counted per grid position. *Darker shades of color* represent more points per grid position, summed for the four individuals. Additional labeled cells were found in more lateral sections (not shown). *Scale bars* **a**, **c** 200 µm; **b** 50 µm

The cumulus does not begin its migration until mesendoderm ingression through the central and annular blastospores is underway and a distinct lower layer has formed. This could mean that the cumulus in *C. mildei* forms by cell–cell interaction within the primitive plate, as postulated by the canonical model. To examine this possibility, we labeled early-ingressing cells with CM-DiI: embryos were injected directly into the nascent deep layer below the early blastopore. During cumulus migration, in both external view and in histological section, label was found in only the cumulus mesenchyme cells (*N* = 15, Fig. 8a, b). If the cumulus cells were to differentiate from a mixed population of cells in the early deep layer, CM-DiI labeling of these cells should yield label in both cumulus and generalized mesendoderm. Our results show that only cumulus cells are labeled by early injection, so specification of cumulus cells likely occurs before general mesendoderm internalization.

#### Fate of the cumulus

Once the cumulus has migrated to the edge of the germ disc or the equivalent position in *C. mildei*, its cells disperse as the dorsal field forms. Until the present study, only Holm [11] was successful in following the fate of cells beyond dorsal field formation by labeling cumulus cells in *A. labyrinthica* with carmine powder. Holm was able to find carmine particles in the opisthosoma, but presented only one case. We raised four injected *C. mildei* embryos to hatching, and all labeled cells were found in the opisthosoma, confirming Holm’s basic finding (Fig. 8c). Most labeled cells were in the dorsolateral region of the abdomen. A limitation of our study is that development in oil (necessary for the injections) interferes with chitinization and molting, and development arrests after hatching. Organogenesis is not complete at this stage, particularly in the opisthosoma; so there is no absolute certainty regarding the ultimate adult fate of the labeled cells. However, histological sections of uninjected controls, including second instar and adult, suggest that the dorso-lateral abdomen is composed largely of gut and gut diverticula (Additional file 9: Figure S4). Therefore, the labeled cells probably form some portion of the digestive mass. This is consistent with molecular data that cumulus cells express endodermal, but not mesodermal markers [17, 18]. It is also possible that the labeled cells are fated to form visceral mesoderm.

## Discussion

**Fig. 9:**
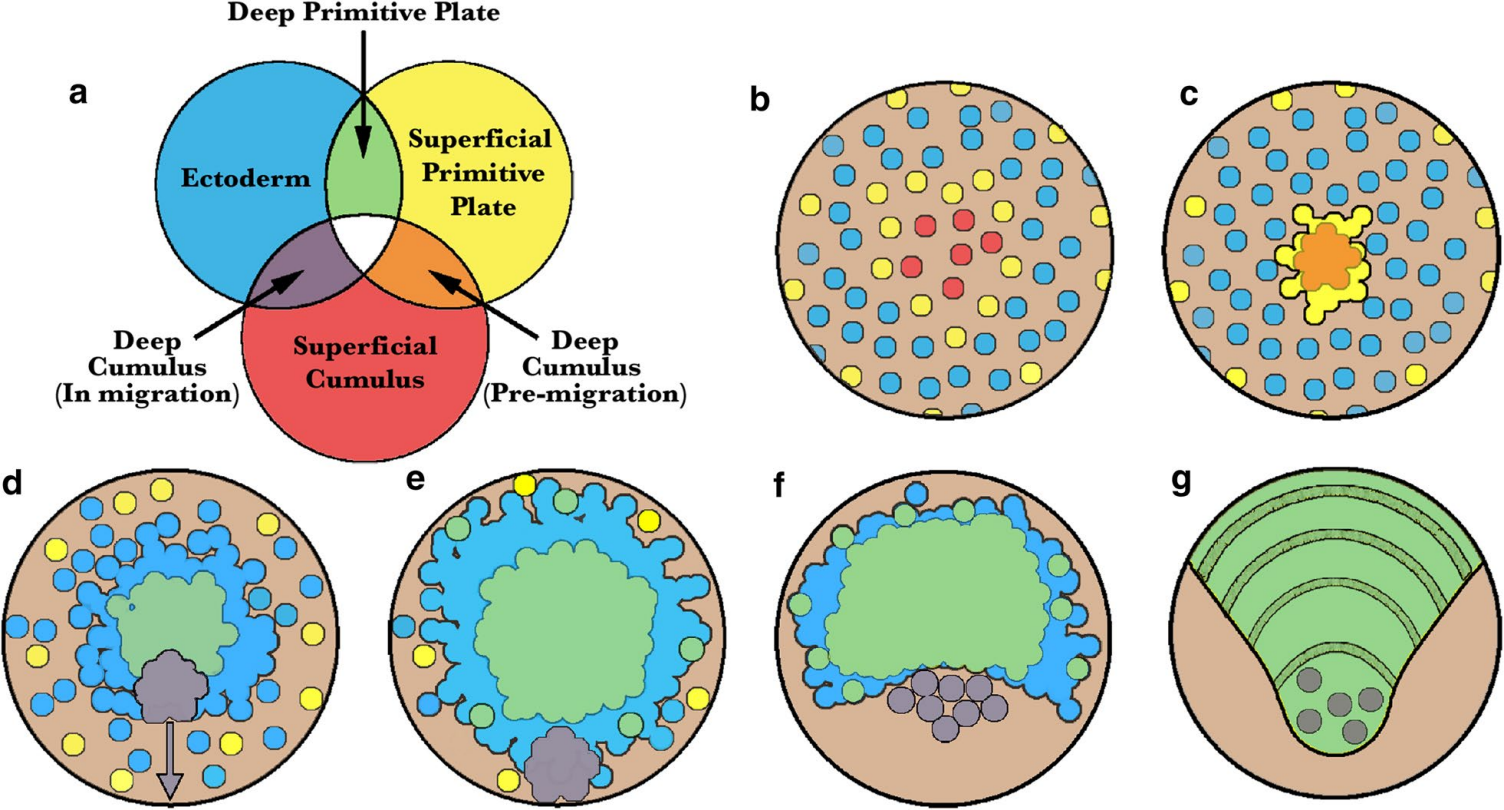
Our new model of spider development showing origin of deep cells and fate of cumulus. **a** Venn diagram representing superficial and deep cells: key to color-coding in **b**–**g.** Before gastrulation, the embryo’s cell types are all on the surface: *red* represents cumulus before internalization; *yellow* represents prospective mesendoderm; *blue* represents ectoderm. During gastrulation, multiple layers form, which are shown as overlays of the colors representing the superficial and deep layers. **b** Pre-gastrula. Cells nearest the central blastopore are fated to become the cumulus (*red*). **c** Gastrulation begins. Cumulus cells internalize as a group (and so appear *orange*, i.e. *red* under *yellow*). **d** Mid gastrula. Other presumptive deep cells have internalized through the central blastopore to form the primitive plate mesendoderm (*green*, i.e., *yellow* under *blue*). **e** Late gastrula. Cumulus has migrated beneath the superficial layer and additional mesendoderm cells internalize at extra-blastoporal sites (individual *green* cells). **f** Dorsal field (extraembryonic epithelium) begins to expand in posterior. Cumulus cells disperse within the deep layer. **g** Germ band has formed. Most cumulus cells have a posterior fate

This paper has used live-cell imaging and labeling techniques to demonstrate that spider gastrulation comprises multiple phases of cell internalization, which occur in more than one region of the embryo. Multi-phasic and multi-regional cell internalization contradicts the canonical model of spider gastrulation, which defines the cumulus and other internalized cells as the product of a single internalization event. The more complex pattern of gastrulation is now known definitively in five species representing three spider families: *L. mactans* and *geometricus, P. tepidariorum* (Theridiidae), *Z. x*-*notata* (Araneidae), and *C. mildei* (Eutichuridae). Based on our data, a new working model of spider development incorporates gastrulation at multiple points. Figure 9 shows superficial and deep layers as color overlays to represent the positions of distinct cell populations in 3-dimensional space throughout early development (Fig. 9a, Venn diagram). As shown in the figure, gastrulation includes successive rounds of cell internalization, in order: (1) cumulus mesenchyme, (2) central primitive plate mesendoderm, and (3) a late, variable contribution to the primitive plate, at the germ disc rim (*Latrodectus* spp.*, P. tepidariorum*); extra-blastoporal ring (*C. mildei*); or central caudal bud (*Z. x*-*notata*). In *P. tepidariorum*, the cells internalizing at the rim express *twist* [29] and presumably are fated as mesoderm. No data are available on gene expression patterns in these cells of other species; and definitive cell fates are unknown in all cases, so we cannot know whether cells internalizing at the germ disc rim in *Latrodectus* spp. and *P. tepidariorum* and the extra-blastoporal ring in *C. mildei* are homologous.

Periodic, static observations of the dynamic process of spider gastrulation by many authors gave valuable information (summarized in [27]), but were unable to distinguish a multi-phase and multi-point process from a single, continuous internalization at a single locus. Whether complex gastrulation is common to all spiders and the extent to which different species rely on complex internalization strategies remains unknown. However, we have summarized from available literature the data on cell internalization across 11 spider genera from 3 sub-orders (Table 1). In the more basal Mesothelae and Mygalomorphae, and in the Araneomorph *Torania*, there appears to be precocious ingression of scattered individual cells at multiple points throughout the blastoderm, apparent as small opaque spots [12, 25, 33–35]. Thus, four distinct cell internalization events have been reported by various authors: (1) early ingression of single cells prior to formation of the blastopore, (2) cumulus-first internalization at the blastopore, (3) internalization at the germ disc rim or extra-blastoporal ring, and (4) late-phase internalization at the caudal end of the nascent germ band. We have not observed early or late-phase cell internalization in *Latrodectus* spp. or *C. mildei* and so our new working model focuses on events 2 and 3 as shown in Figs. 2 and 9.

**Table 1:** Evaluation of cell internalization traits across spider phylogeny

Sub order	Genus	Source	Trait			
			Ingression of single cells before blastopore forms	Cumulus forms before Primitive Plate	Internalization at germ disc rim or extra-blastoporal site	Late internalization at caudal structure
**Models**	***Canonical model***	[11, 12]	No	No	No	No
	***Working model***	*This paper*	No	***Yes***	***Yes***	No
***Mesothelae***	*Heptathela*	[33]	*Yes*	*No cumulus?*	***Yes***	No
***Mygalomorphae***	*Atypus*	[34]	*Yes*	No	No	No
	*Ischnothele*	[12]	*Yes*	No	No	*Pit present* ^a^
	*Ischnocolus*	[35]	*Yes*	No	No	No
***Araneomorphae***	*Agelena*	[12, 13]	No	No	No	*Pit present* ^a^
	*Torania*	[25]	*Yes*	No	No	No
	*Cupiennius*	[21]	No	No	No	No
	*Cheiracanthium*	*This paper*	No	***Yes***	***Yes***	No
	*Latrodectus*	*This paper*	No	***Yes***	***Yes***	No
	*Parasteatoda*	[17, 29]	No	No	***Yes***	*Pit present* ^a^
	*Zygiella*	[26]	No	***Yes***	No	*Yes*

Bold italic terms indicate agreement with our model

Italic terms indicate agreement with canonical model but data are not incorporated into our model

Plain text terms indicate agreement with canonical model

^a^Presence of a pit suggests cell internalization, but direct evidence (cell tracing, histology) does not exist

The early gastrulation pattern in *C. salei* (Ctenidae) appears to follow the canonical model, in that an early deep layer forms first (‘primary thickening’), from which the cumulus emerges [21], but is there bona fide internalization at the periphery? Does the cumulus in *P. tepidariorum* internalize separately from other deep layer cells? Applying our methods to these species would help evaluate our working model. Definitive evidence for late internalization at a caudal structure exists only for *Z. x*-*notata*, in which a large group of cells is internalized through a structure termed the ‘caudal bud’ [26]. In *A. labyrinthica*, *I. karschi* and *P. tepidariorum*, a pit is present at the vertex of the expanding dorsal field [12, 13, 17]. The presence of a pit would seem to indicate internalization; however, there is no direct evidence for caudal internalization in these species.

How unusual is gastrulation at multiple times and positions in the embryo? We are perhaps used to thinking of a single blastoporal region that functions continuously in such model systems as the frog with its annular blastopore or the chick and mouse with a linear primitive streak. There are, however, many examples of embryos with temporally and spatially separate points of internalization. The primary mesenchyme cells of many sea urchins ingress singly or in small groups hours before internalization of the archenteron, and a secondary invagination of the stomodeum completes the through-gut (reviewed in [36]). The fruit fly internalizes its mesoderm by invagination of the ventral furrow long before the anterior and posterior midgut invaginations at opposite ends of the egg move prospective endoderm into the interior. This pattern of separated mesoderm and endoderm internalization holds for many other insects [37]. Similarly, gastrulation in non-malacostracan crustaceans often involves internalization of mesoderm and endoderm separated in time, space, or both; and that of malacostracans also involves early ingression followed by mass internalization of mesendoderm [38]. Thus, spatial and temporal separation of internalizing cell populations is not unusual, particularly in groups with relatively early specification of cell fate.

Observations of living embryos without experimental challenge cannot answer questions of cell fate determination. Nevertheless, we believe our results are consistent with the view that the cells of the cumulus are fated as such before their internalization. Timelapse videos show that the cumulus cells ingress first in all species we studied, including *Z. x*-*notata* [26], and the identity of these early ingressors is confirmed by CM-DiI injection in *L. mactans* and *C. mildei*: only cumulus mesenchyme cells are labeled by early injection into the deep layer. The cumulus cells, thus, exhibit a unique behavioral phenotype that correlates with a unique molecular phenotype (*dpp* expression in *P. tepidariorum* [17]). Both observations challenge the canonical view that the cumulus cells differentiate by cell–cell interaction within the deep layer. Our tracings and classic experiments by Holm suggest an endodermal fate for the cumulus [11], consistent with expression in central deep cells of *forkhead* [18], a marker of early endoderm in *Drosophila*. A more complete understanding of the state of determination of the cells of the early spider gastrula will require cell ablation and transplant studies.

This paper has emphasized the embryology of early spider development to reveal that the canonical model of spider gastrulation is not accurate for all species. Are the data potentially useful in understanding other aspects of spider biology? The variation in gastrulation strategies among the different species (summarized in Table 1) may be of value in clarifying phylogenetic relationships. The monophyly of the orb-weavers (Orbiculariae), which includes *Z. x*-*notata* (Araneidae) and the cobweb-weavers *P. tepidariorum* and *Latrodectus* spp. (Theridiidae), is methodologically disputed [39, 40]. The reason for this dispute is that the characters which establish the monophyly are almost entirely behavioral [41], while molecular data are conflicted [42, 43], but tend not to support monophyly of the Orbiculariae [44–46]. However, our data suggest that the Orbiculariae may be united by shared developmental similarities that vary from the canonical model, namely early cumulus ingression and late cell internalization at an extra-blastoporal site. The three spiders whose development seems most similar to the canonical model are all members of the RTA clade (a diverse Entelegyne lineage) [47, 48]: *A. labyrinthica* (Agelenidae), *C. salei* (Ctenidae), and *C. mildei* (Eutichuridae, assigned by [49]). Most phylogenies place the RTA clade as sister group to the Orbiculariae [43]. Wolff and Hilbrant [21] report that gastrulation in *C. salei* largely follows the canonical model; however, we found notable variation from the model in *C. mildei* that could not be easily detected without tracing individual cells. Our data from *C. mildei* are the first evidence of multi-phase gastrulation outside the Orbiculariae. Evidence (or evidence of absence) for multi-phase gastrulation should be sought in the popular spider systems, *C. salei* and *P. tepidariorum,* and in the historical spider model, *A. labyrinthica*, to help clarify these evolutionary relationships.

## Conclusions

Our cell tracings and those of [26, 29] show that gastrulation in spiders typically involves cell internalization at both a central blastopore and, at varying later times, internalization at an extra-blastoporal site. Internalization occurs at the peripheral rim of the germ disc in *P. tepidariorum* and *Latrodectus* spp.; at an annulus distinct from the central blastopore in *C. mildei*; and at the caudal bud in *Z. x*-*notata*. In contrast, there is presently no evidence for extra-blastoporal internalization in *C. salei* or the historical model *A. labyrinthica*, although the latter has not been studied with modern methods. Our working model shown in Figs. 2 and 9 reflects our view that multiple regions of cell internalization are typical for spiders.

Live-cell labeling with CM-DiI and cell tracings demonstrate that cells of the prospective cumulus (dorsal organizer) internalize first, well before general mesendoderm ingresses into the deep layer. Injection of CM-DiI into the deep layer beneath the central blastopore early in gastrulation labels only cumulus mesenchyme cells. We show that these cells’ daughters come to populate the deep endoderm of the opisthosoma, confirming Holm’s work in *A. labyrinthica.* After the cumulus has begun migration, CM-DiI injection into the same region labels non-cumulus mesoderm and endoderm. Although live labeling cannot definitively address the state of cell determination in any system, the early ingression of the prospective cumulus as a distinct event in gastrulation is consistent with early, cell-autonomous specification of this cell type. Cell–cell interaction within the deep layer of the primitive plate is unlikely to be required for formation of the cumulus, as the cumulus begins to migrate in many species before the deep layer contains many other cells. Our working model incorporates early internalization and cell-autonomous formation of the cumulus.

The demonstration of multiple sites of gastrulation in spiders accords with data from many other arthropod systems, from crustaceans to flies. Multiple gastrulation sites may not be universal among spiders, however, and a wider sampling of spider taxa may reveal systematic variation that could be useful in establishing phylogenetic relationships. Another character of possible utility is origin of the cumulus, which could form either by cell-autonomous mechanisms or by cell–cell interaction within an undifferentiated deep layer.

## Additional files

10.1186/s13227-015-0029-z Timing of key developmental stages. Durations of signal stages (hours) in *Cheiracanthium mildei* and *Latrodectus mactans*.
10.1186/s13227-015-0029-z Signal stages of *Latrodectus mactans* embryos. Abbreviations: BP, blastopore; C, cumulus; PP, primitive plate; VS, ventral sulcus. Bars in ventral sulcus show its width. Blue dots mark position of leg rudiments at their base in the lateral germ bands. Scale bar: 200 µm.
10.1186/s13227-015-0029-z Signal stages of *Cheiracanthium mildei* embryos. Abbreviations: BP, blastopore; C, cumulus; PP, primitive plate; VS, ventral sulcus. Bars in ventral sulcus show its width. Blue dots mark position of leg rudiments at their base in the lateral germ bands. Scale bar: 200 µm.
10.1186/s13227-015-0029-z Two-phase gastrulation in ***Latrodectus mactans***. Prospective cumulus cells (false-colored red and pink) internalize first at the central blastopore. Other prospective deep layer cells (yellow and orange) internalize later to form generalized mesendoderm. Yellow cells at germ disc rim will internalize later. Blue cells are examples of cells known to persist in the superficial layer as long as their daughter cells can be visualized. Elapsed time ~34 hr. Same embryo as ‘Lm embryo 1’ in Fig. 3.
10.1186/s13227-015-0029-z Two-phase gastrulation in ***Latrodectus mactans***. Prospective cumulus cells (false-colored red and pink) internalize first at the central blastopore. Other prospective deep layer cells (yellow and orange) internalize later to form generalized mesendoderm. Yellow cells at germ disc rim will internalize later. Blue cells are examples of cells known to persist in the superficial layer as long as their daughter cells can be visualized. Dots indicate positions of cells that become visible during the movie. Elapsed time ~21 hr. Same embryo as ‘Lm embryo 2’ in Fig. 3.
10.1186/s13227-015-0029-z Internalization at the rim of the germ disc in ***Latrodectus mactans***. Cells false-colored yellow ingress at the rim of the germ disc. Blue cells are examples of cells known to persist in the superficial layer as long as their daughter cells can be visualized. Elapsed time ~20 hr. Same embryo as ‘Lm embryo 1’ in Figs. 3 and 6.
10.1186/s13227-015-0029-z Internalization at the rim of the germ disc in ***Latrodectus geometricus***. Cells false-colored yellow ingress at the rim of the germ disc. Blue cells are examples of cells known to persist in the superficial layer as long as their daughter cells can be visualized. Elapsed time ~20 hr. Same embryo as ‘Lg embryo 1’ in Fig. 6.
10.1186/s13227-015-0029-z Two-phase gastrulation at two loci in ***Cheiracanthium mildei***. Prospective cumulus cells (false-colored red and pink) internalize first at the central blastopore. Other prospective deep layer cells (yellow and orange) internalize later at a separate ring outside the blastopore to form mesendoderm. Elapsed time ~21 hr. Same embryo as ‘Embryo 1’ in Fig. 7.
10.1186/s13227-015-0029-z ***Cheiracanthium mildei*** in parasagittal sections selected to show position and degree of differentiation of gut and its diverticula. Posterior at right. A, Second instar juvenile. Red outline shows position of cumulus cells revealed in separate experiments with DiI labeling, please see Fig. 8. Note relatively undifferentiated opisthosoma; gut is incomplete and many yolk particles persist (stained orange). B, Adult, section through left side showing gut diverticula filling lateral abdominal cavity. C. Adult, section through midline of same spider showing patent midgut with connection to dorsal and ventral diverticula. Scale bars: A, 200 µm; B, C: 1 mm.

## Abbreviations

BP: blastopore
C: cumulus (dorsal organizer)
CM-DiI: 1,1′-dioctadecyl-3,3,3′,3′-tetramethylindocarbocyanin perchlorate
DAPI: 4′,6-diamidino-2-phenylindole
DF: dorsal field
DMSO: dimethyl sulfoxide
Lm: *Latrodectus mactans*
Lg: *Latrodectus geometricus*
PBS: phosphate-buffered saline
PP: primitive plate
PTD: 0.1 % Triton + 5 % DMSO in PBS
RTA: retrolateral tibial apophysis
VS: ventral sulcus
