# Supplementary figures and images for: Gastrulation occurs in multiple phases at two distinct sites in Latrodectus and Cheiracanthium spiders
Source: EvoDevo. 2015 Oct 21;6:33. doi: 10.1186/s13227-015-0029-z (PMC4618530; doi:10.1186/s13227-015-0029-z)

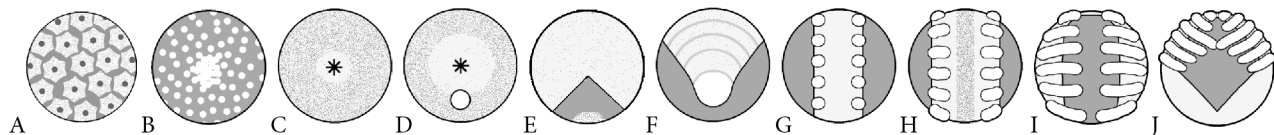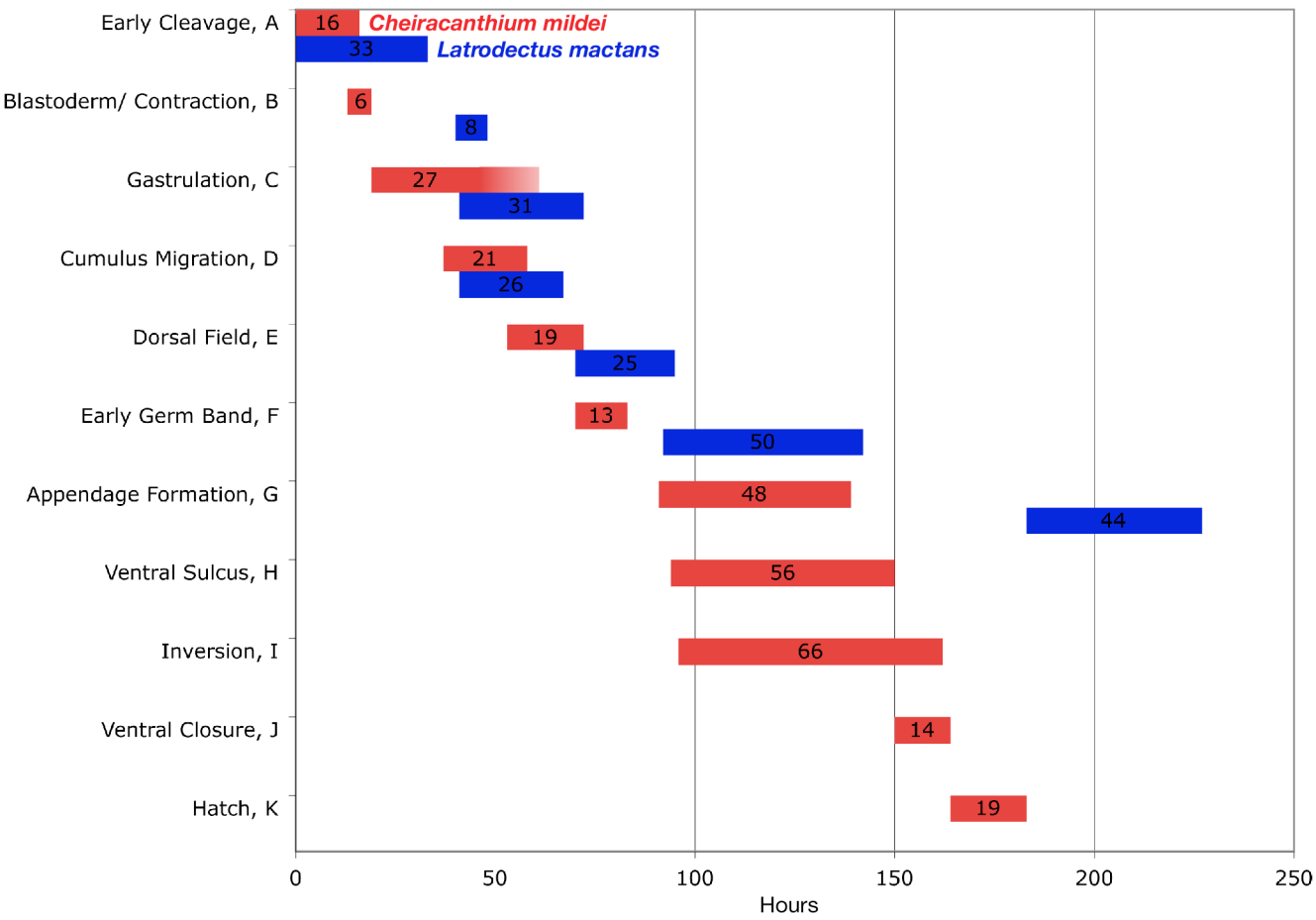

Supplement: Supplementary file 1 — 10.1186/s13227-015-0029-z Timing of key developmental stages. Durations of signal stages (hours) in Cheiracanthium mildei and Latrodectus mactans. [file 13227_2015_29_MOESM1_ESM.pdf]

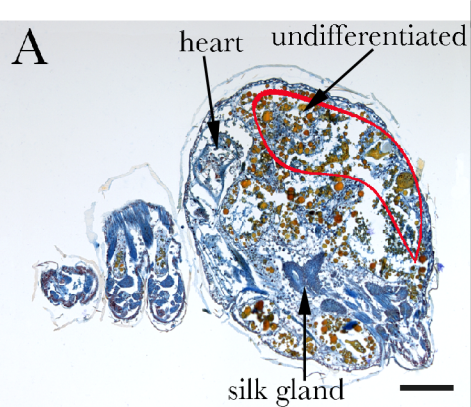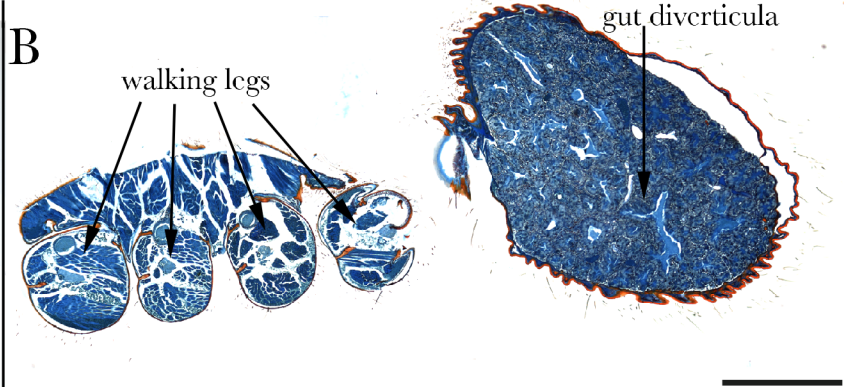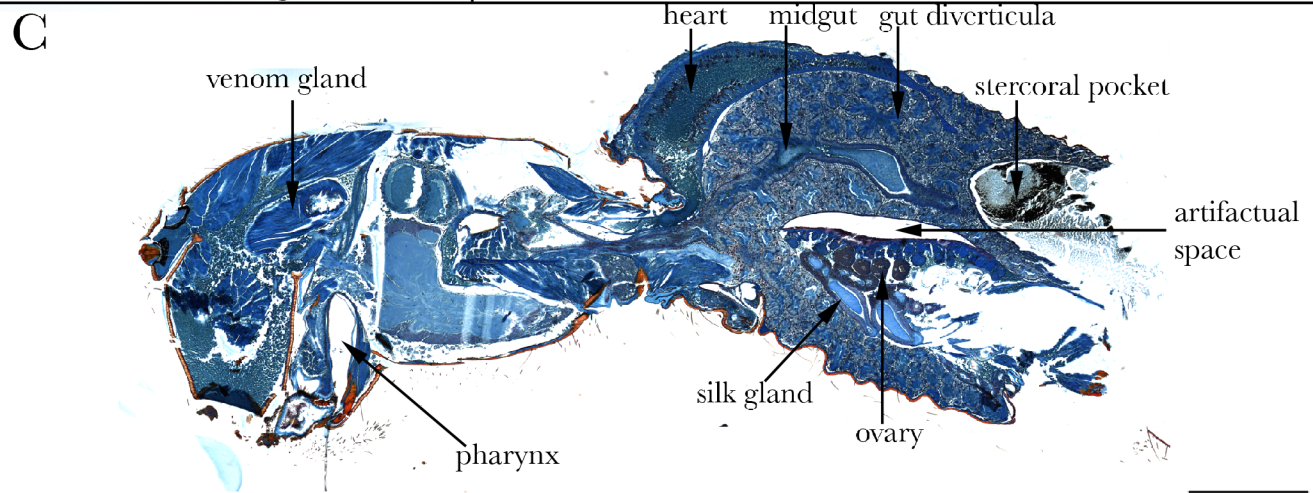

Supplement: Supplementary file 4 — 10.1186/s13227-015-0029-z Two-phase gastrulation in Latrodectus mactans. Prospective cumulus cells (false-colored red and pink) internalize first at the central blastopore. Other prospective deep layer cells (yellow and orange) internalize later to form generalized mesendoderm. Yellow cells at germ disc rim will internalize later. Blue cells are examples of cells known to persist in the superficial layer as long as their daughter cells can be visualized. Elapsed time ~34 hr. Same embryo as ‘Lm embryo 1’ in Fig. 3. [file 13227_2015_29_MOESM4_ESM.pdf]
