# Supplementary material for: Gastrulation occurs in multiple phases at two distinct sites in Latrodectus and Cheiracanthium spiders
Source: EvoDevo. 2015 Oct 21;6:33. doi: 10.1186/s13227-015-0029-z (PMC4618530; doi:10.1186/s13227-015-0029-z)
Supplement: Supplementary file 2 — 10.1186/s13227-015-0029-z Signal stages of Latrodectus mactans embryos. Abbreviations: BP, blastopore; C, cumulus; PP, primitive plate; VS, ventral sulcus. Bars in ventral sulcus show its width. Blue dots mark position of leg rudiments at their base in the lateral germ bands. Scale bar: 200 µm. [file 13227_2015_29_MOESM2_ESM.pdf]

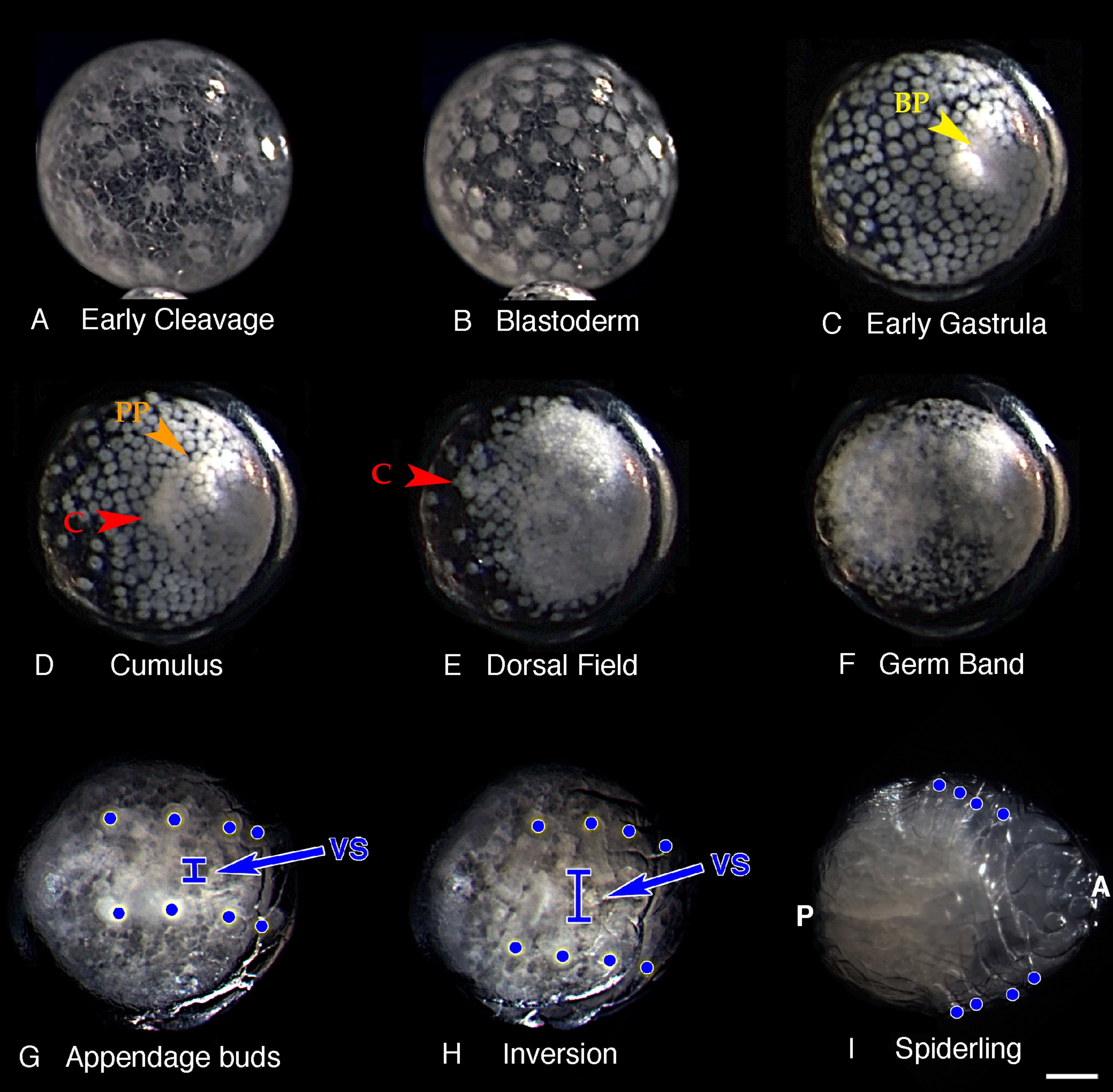
A Early Cleavage
B Blastoderm
C Early Gastrula
BP
D Cumulus
PP
C
E Dorsal Field
C
F Germ Band
G Appendage buds
VS
H Inversion
VS
I Spiderling
P
A
