# Supplementary material for: Gastrulation occurs in multiple phases at two distinct sites in Latrodectus and Cheiracanthium spiders
Source: EvoDevo. 2015 Oct 21;6:33. doi: 10.1186/s13227-015-0029-z (PMC4618530; doi:10.1186/s13227-015-0029-z)
Supplement: Supplementary file 3 — 10.1186/s13227-015-0029-z Signal stages of Cheiracanthium mildei embryos. Abbreviations: BP, blastopore; C, cumulus; PP, primitive plate; VS, ventral sulcus. Bars in ventral sulcus show its width. Blue dots mark position of leg rudiments at their base in the lateral germ bands. Scale bar: 200 µm. [file 13227_2015_29_MOESM3_ESM.pdf]

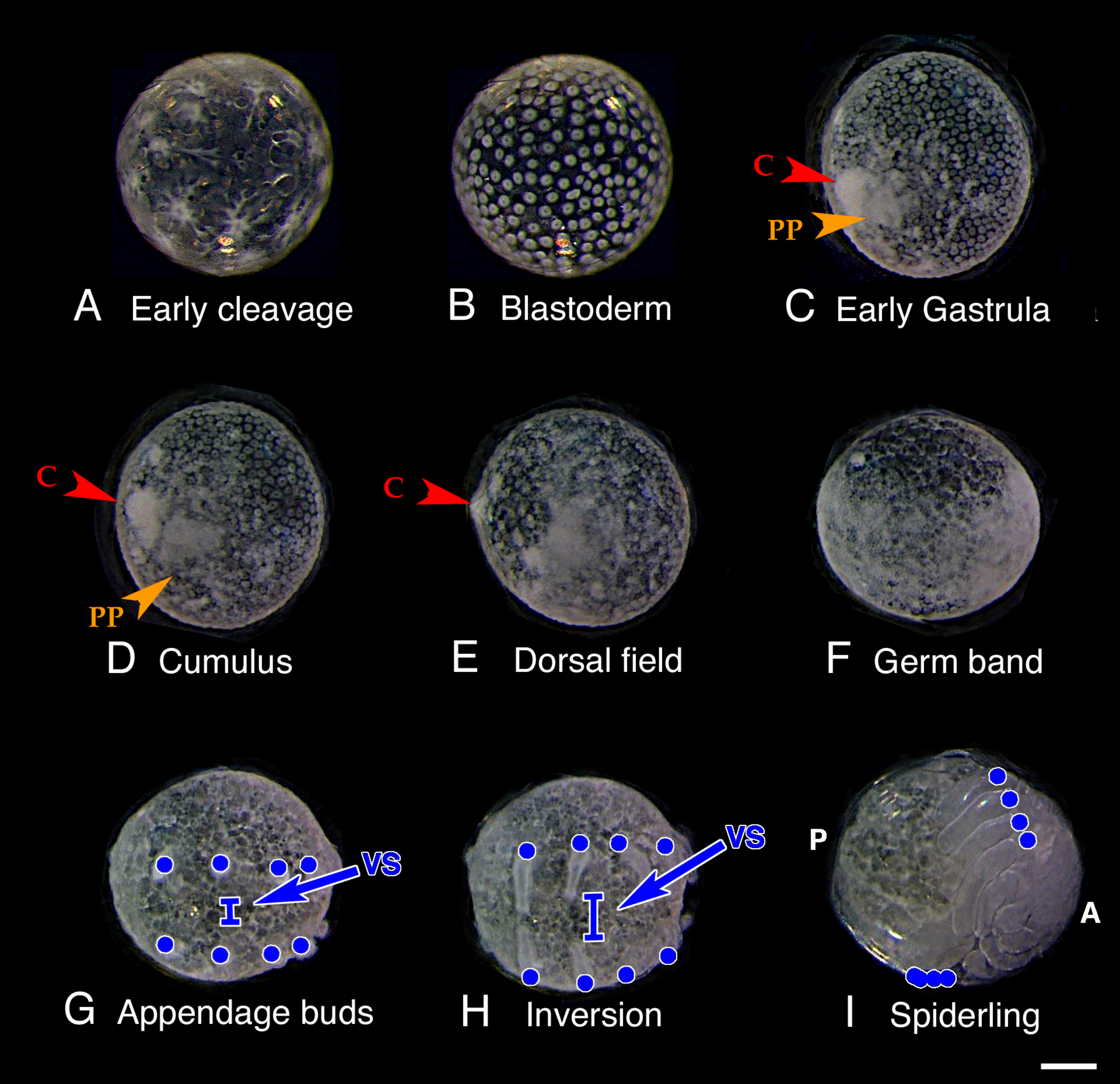

A Early cleavage
B Blastoderm
C
PP
C Early Gastrula
C
PP
D Cumulus
C
E Dorsal field
F Germ band
VS
I
G Appendage buds
VS
I
H Inversion
P
A
I Spiderling
